# Infected intradural dermoid cyst without dermal sinus tract mimicking brain abscess: A case report

**DOI:** 10.1016/j.ijscr.2020.05.052

**Published:** 2020-05-30

**Authors:** Anh Hoang Pham, Tam Duc Le, Hung Thanh Chu, Tuan Anh Le, Ha Dai Duong, He Van Dong

**Affiliations:** a Neurosurgery Department I, Viet Duc University Hospital, Hanoi, Viet Nam; b Hanoi Medical University, Hanoi, Viet Nam; c Neurosurgery Center, Viet Duc University Hospital, Hanoi, Viet Nam

**Keywords:** Infection, Intradural dermoid cyst, No dermal sinus, Children

## Abstract

**Introduction:**

Infection is a rare complication of intradural dermoid cyst. We reported an infected intradural dermoid cyst without dermal sinus tract mimicking brain abscess.

**Presentation of Case:**

A 4-year-old boy with no medical history complained of a palpable mass on his head. On examination, the occipital palpable mass was firm and immobile, had no redness, swelling, and pain. The preoperative MRI showed a well-defined, ring-enhancing lesion. White blood count was $12.8\;\times \;10^{9}\;$ cells/liter. We have sought no other infection sites. We encountered intraoperatively the pus from the infected mass invading subcutaneous layer and skull bone. We excised completely the tumor and carefully coagulated the residual capsule invading superior sagittal sinus. Histopathological examination was infected dermoid cyst. The infection agent was *Staphylococcus aureus.* The patient was received systemic antibiotic therapy for 21 days following oral antibiotics for 1 month. He was discharged with no complications.

**Discussion:**

The diagnosis of infected dermoid cyst was often based on MRI images and especially dermal sinus tract. However, in the absence of a dermal sinus tract, preoperative diagnosing an infected dermoid cyst might be very challenging. The ideal treatment of the dermoid cyst was total resection of the cyst with the epithelial lining. Nevertheless, due to the benign nature of dermoid cyst, adequate gross total resection with meticulous hemostasis the residual capsule firmly adhering eloquent areas and major vessels was more appropriate.

**Conclusion:**

Diagnosing infected dermoid cyst with no dermal sinus might be challenging. Systemic antibiotic therapy after gross total resection was an effective treatment.

## Highlights

• An infected intradural dermoid cyst with no dermal sinus was exceptional.
• Diagnosing infected dermoid cyst with no dermal sinus might be challenging.
• Systemic antibiotic therapy after gross total resection was an effective treatment.

## 1 Introduction

An intracranial dermoid cyst is a benign, ectopic squamous epithelial cyst, including elements of the dermis, such as hair follicles, sebaceous glands, and sweat glands. This is a rare lesion and accounts for 0.3 % of intracranial tumors [1]. Regarding etiology, two embryogenic theories were widely accepted. Firstly, this cyst was derived from retained surface ectoderm trapped by two fusing neuroectodermal surfaces during neural tube closure. Secondly, this due to the inclusion of cutaneous ectoderm at the time of neural tube closure, 3rd to 5th week of embryogenesis [2]. Total resection of the cyst with the epithelial lining was the ideal treatment.

Infection was a rare complication of the dermoid cyst and was usually associated with dermal sinus tract [[3], [4], [5]]. The most common agent was *Staphylococcus aureus* [6]. Preoperative diagnosing an infected dermoid cyst without the dermal sinus tract might be very challenging. This might mimic brain abscess. In this paper, we reported an infected intradural dermoid cyst without the dermal sinus tract mimicking brain abscess. This is the first case report on this issue in Vietnam.

The work has been reported in line with the SCARE criteria [7].

## 2 Presentation of case

A 4-year-old boy with no medical history complained of a palpable mass on his head. He had no headache, nausea, vomiting and blurred vision. On examination, he was alert, oriented. The occipital palpable mass was firm and immobile, had no redness, swelling, and pain. He had a normal temperature. He denied paralysis and cranial nerve palsies.

**Fig. 1:**
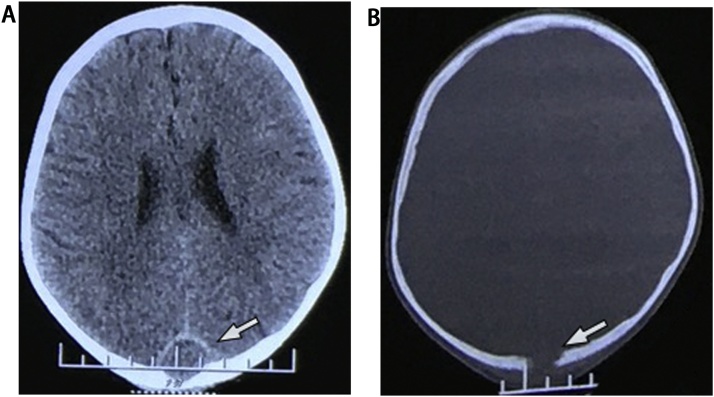
On CT scan, an occipital well-defined isodensity round mass had a thin enhanced peripheral rim, invaded skull bone, and superior sagittal sinus.

**Fig. 2:**
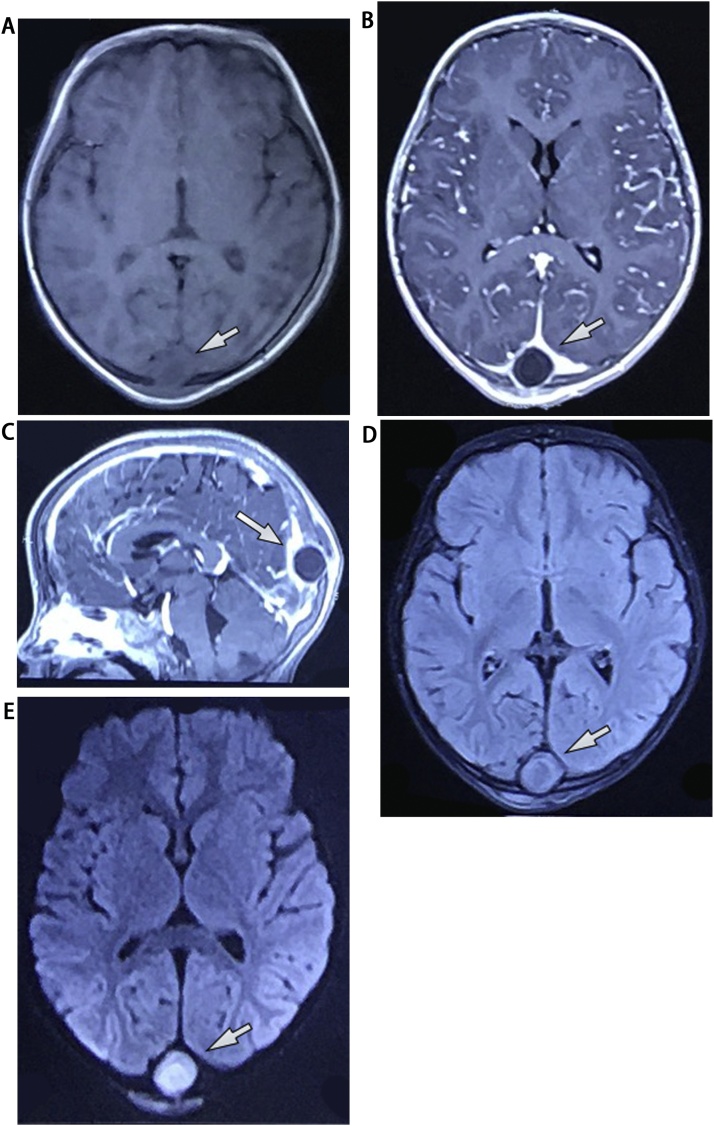
On preoperative MRI, an extra-axial isointense mass on T1W enhanced peripherally and invaded superior sagittal sinus on gadolinium-enhanced T1W, was hyperintense on FLAIR and hypoperfused on DWI.

The preoperative CT scan showed an occipital well-defined isodensity round mass, which had a thin peripheral rim enhancement, invaded skull bone and superior sagittal sinus (SSS) (Fig. 1). The preoperative MRI showed an extra-axial isointense mass on T1W, which enhanced peripherally on gadolinium-enhanced T1W. This mass was hyperintense on FLAIR and restricted diffusion on diffusion-weighted imaging (DWI) (Fig. 2). On complete blood count, white cell count was $12.8\;\times \;10^{9}\;$ cells/liter, and absolute neutrophils were $7.82\;\times \;10^{9}\;$ cells/liter (60.9 %). Erythrocyte sedimentation rate (ESR) was increased with 74 mm after one hour. We have sought no infection site on skin, ENT, dental, genitourinary and pulmonary systems. The preoperative diagnosis was brain abscess.

**Fig. 3:**
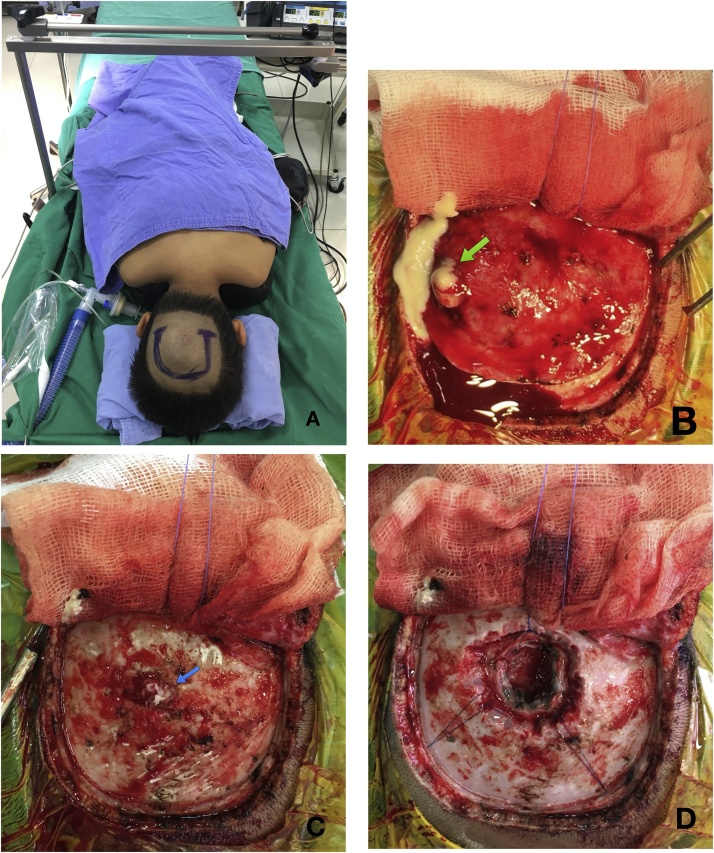
(A) the patient was in the prone position and U-shaped incision was used. (B) The pus (green arrow) was taken and sent for culture. (C) The skull bone was invaded (blue arrow). (D) Operative field after opening dura and tumor excision.

**Fig. 4:**
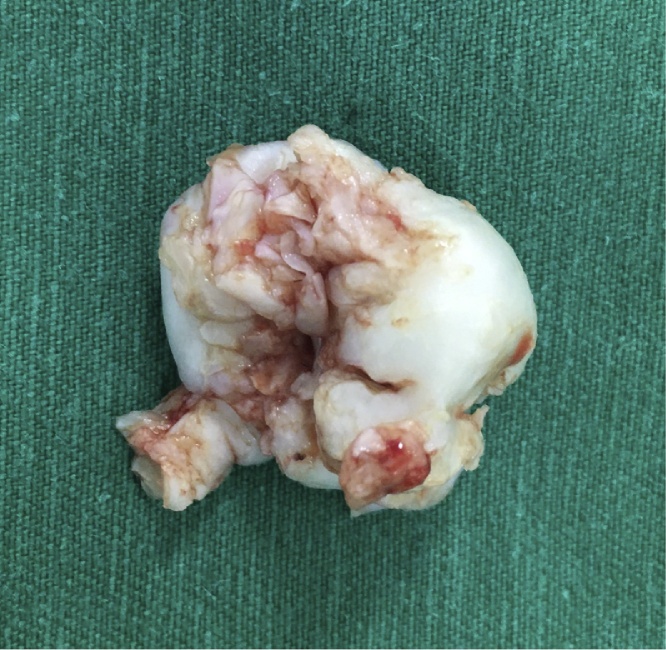
On macroscopic pathology, the tumor was a well-defined, lobulated, pearly mass.

The occipital approach craniotomy was used with a U-shaped incision. After the elevation of the skin flap, we encountered the pus from the infected mass invading the skull bone and the subcutaneous layer (Fig. 3). The pus was taken and sent for culture. We expanded the invasion of skull bone and dura. The tumor was a well-defined, lobulated, pearly mass (Fig. 4). We excised completely tumor and carefully coagulated the residual capsule invading SSS. The operative field was irrigated and debrided meticulously with saline and povidone-iodine solution and it was injected gentamicin. The infected bone flap was removed. Histopathological examination was infected dermoid cyst

**Fig. 5:**
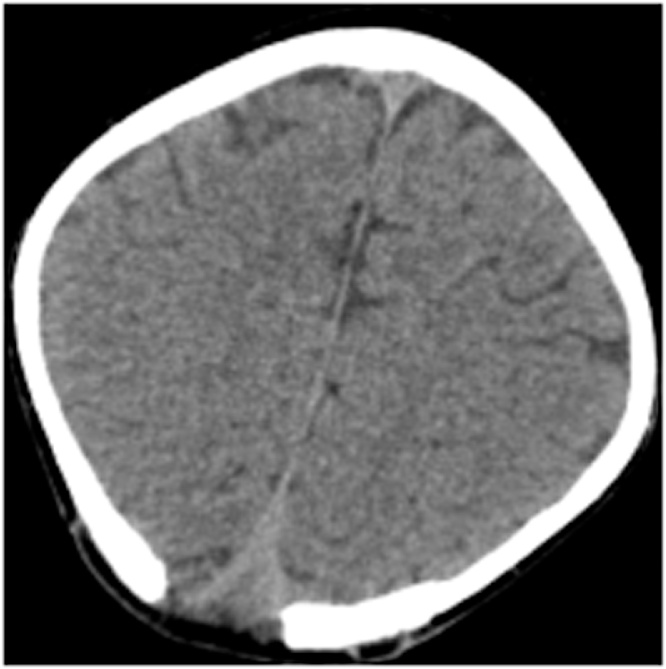
At a one-month postoperative follow-up, CT scan shows no remnant mass.

After operation, the patient was administered empiric systemic antibiotic therapy (ceftriaxone 100 mg/kg IV single dose + metronidazole 30 mg/kg/day IV divided q6h + vancomycin 15 mg/kg IV q8h). The bacterial culture result was *Staphylococcus aureus*, which was sensitive to ceftriaxone, vancomycin, clindamycin, co-trimoxazole, and erythromycin. The patient was received systemic antibiotic therapy for 21 days following oral antibiotics for 1 month. He was discharged with no complications and neurosurgical deficits. At a one-month postoperative follow-up, the CT scan showed no remnant mass (Fig. 5).

## 3 Discussion

Rarely, an intracranial dermoid cyst can be infected. If so, the diagnosis was often based on SIRS, MRI images and especially dermal sinus tract [[3], [4], [5]]. However, in the absence of a dermal sinus tract, preoperative diagnosing an infected dermoid cyst might be a great challenge. In our case, the 4-year-old boy had no signs and symptoms of infection. The only clue of infection was high ESR and a hypointense mass on T1W with rim enhancement and restricted diffusion on DWI. We found no primary infection site on the skin, ENT, dental, genitourinary and pulmonary systems. Our preoperative diagnosis was brain abscess. The infected dermoid cyst was only confirmed by histopathological examination. Similarly, Diyora et al. reported a 3-year-old child with an infected spinal dermoid cyst without a sinus tract in 2019. His preoperative diagnosis was conus ependymoma [8] (Fig. 5).

The ideal treatment of the dermoid cyst was total resection of the cyst with the epithelial lining. Nevertheless, due to the credo of medicine "Primum non-nocere" meaning "First, do no harm" and benign nature of dermoid cyst, adequate gross total resection with meticulous hemostasis the residual capsule firmly adhering eloquent areas and major vessels was appropriate. Moreover, the risk of recurrence and neurological deficit might be reduced by carefully bipolar coagulation [9,10]. In the case of the infected dermoid cyst, we irrigated meticulously the operative field with saline and povidone-iodine solution. We injected gentamicin to treat the local infection. Tekkök et al used an amikacin solution to rinse the operative field [6]. Although *Staphylococcus aureus* was the most common infected pathogen [6,8,11], other agents were also reported such as *Staphylococcus epidermidis* [3]*, Escherichia coli* [12]*, anaerobes* [13]. The sources of infection were dermal sinus tract, hematogenous agent spreading from other infection sites, direct invading of adjacent infection, penetrating trauma, or after an operation, and cryptogenic mechanisms.

## 4 Conclusion

Preoperative diagnosing an infected dermoid cyst without the dermal sinus tract might be a great challenge. Systemic antibiotic therapy after adequate gross total resection with meticulous hemostasis of residual capsule invading sinus and eloquent areas was an effective treatment of infected dermoid cysts.

## 5 Funding

This research did not receive any specific grant(s) from funding agencies in the public, commercial, or not-for-profit sectors.

## Ethical approval

Nothing to declare, as this is a single case report. At our center, we do not require ethical review by the Institutional Review Board for single case report studies.

## Consent

Written informed consent was obtained from his parents, for publication of this case report and accompanying images. A copy of the written consent is available for review by the

Editor-in-Chief of this journal on request.

## Registration of research studies

Not applicable – this is a single case report, not a systematic review or meta-analysis. Moreover, we attest that it is not a ‘first in man’ study, either.

## Guarantor

Anh Hoang Pham

## Provenance and peer review

Not commissioned, externally peer-reviewed

## CRediT authorship contribution statement

**Anh Hoang Pham:** Conceptualization, Methodology, Investigation, Writing - original draft, Writing - review & editing. **Tam Duc Le:** Conceptualization, Methodology, Validation, Investigation, Writing - original draft, Writing - review & editing, Visualization. **Hung Thanh Chu:** Investigation, Visualization, Resources. **Tuan Anh Le:** Investigation, Visualization, Resources. **Ha Dai Duong:** Conceptualization, Writing - review & editing, Supervision. **He Van Dong:** Conceptualization, Writing - review & editing, Supervision.

## Declaration of Competing Interest

Nothing to declare.
